# DNA Damage Stress and Inhibition of Jak2-V617F Cause Its Degradation and Synergistically Induce Apoptosis through Activation of GSK3β

**DOI:** 10.1371/journal.pone.0027397

**Published:** 2011-11-08

**Authors:** Toshikage Nagao, Gaku Oshikawa, Nan Wu, Tetsuya Kurosu, Osamu Miura

**Affiliations:** Department of Hematology, Graduate School of Medical and Dental Sciences, Tokyo Medical and Dental University, Tokyo, Japan; University of Barcelona, Spain

## Abstract

The cytoplasmic tyrosine kinase Jak2 plays a crucial role in cytokine receptor signaling in hematopoietic cells. The activated Jak2-V617F mutant is present in most cases of BCR/ABL-negative myeloproliferative neoplasms and constitutively activates downstream signals from homodimeric cytokine receptors, such as the erythropoietin receptor (EpoR). Here we examine the effects of DNA damage stress on Jak2 or Jak2-V617F and on induction of apoptosis in hematopoietic cells. Etoposide or doxorubicin dose-dependently decreased the expression level of Jak2 in UT7 or 32D cells expressing EpoR in the absence of Epo and that of exogenously expressed Jak2-V617F in UT7 cells when cotreated with the Jak2 inhibitor JakI-1 or AG490. Studies with pharmacological inhibitors and genetic manipulations further showed that downregulation of the PI3K/Akt pathway leading to the activation of GSK3β may be involved in downregulation of Jak2 or Jak2-V617F as well as in synergistic induction of Bax activation and apoptosis. The downregulation of Jak2 was inhibited by the proteasome inhibitor MG132 or by expression of both of loss-of-function mutants of c-Cbl and Cbl-b, E3 ubiquitin ligases which facilitated ubiquitination of Jak2-V617F when co-expressed in 293T cells. The pan-caspase inhibitor Boc-d-fmk also inhibited the Jak2 downregulation as well as appearance of a 100-kDa fragment that contained the N-terminal portion of Jak2 in response to DNA damage. Together, these data suggest that DNA damage stress with simultaneous inhibition of the kinase activity causes degradation of Jak2 or Jak2-V617F by caspase cleavage and proteasomal degradation through GSK3β activation, which is closely involved in synergistic induction of apoptosis in hematopoietic cells.

## Introduction

The cytoplasmic tyrosine kinase Jak2 couples with a variety of cytokine receptors, such as the erythropoietin (Epo) receptor (EpoR) and the IL-3 receptor, and plays a crucial role in regulation of proliferation and apoptosis of hematopoietic cells by activating various signaling pathways including the STAT5, RAS/Raf-1/MEK/Erk, and phosphatidylinositol 3'-kinase (PI3K)/Akt pathways [1], [2]. The serine/threonine kinase glycogen synthase kinase-3β (GSK3β) is constitutively active in cells and is regulated through inhibitory phosphorylation on S9 mainly by Akt [3]. GSK3β plays an important role in regulation of protein stability and is involved in regulation of a wide range of cellular processes, ranging from glycogen metabolism to cell-cycle regulation and apoptosis [3], [4], [5]. We have previously shown that a Jak2 inhibitor, Jak inhibitor-I (JakI-1) [6], activated GSK3β by inactivating the PI3K/Akt pathway to phosphorylate cyclin D2 on T280, which triggered its degradation through the ubiquitin proteasome pathway leading to cell cycle arrest of Epo- or IL-3-dependent hematopoietic cells [7]. The somatic valine-to-phenylalanine mutation in the pseudokinase domain of Jak2 (Jak2-V617F) has been found in the majority of patients with polycythemia vera and in about 50% of patients with the other myeloproliferative neoplasms, essential thrombocythemia and primary myelofibrosis [1], [8]. Jak2-V617F is constitutively activated without cytokine stimulation and when coexpressed with homodimeric cytokine receptors, such as EpoR, activates the various downstream pathways leading to cytokine-independent hematopoietic cell proliferation.

Chemotherapeutic agents, including the topoisomerase II inhibitor etoposide and the anthracycline doxorubicin, mostly damage DNA and activate the intrinsic apoptotic pathway leading from Bax activation to mitochondrial damage and caspase activation [9]. We previously found that Epo or IL-3 significantly inhibited etoposide-induced apoptosis in hematopoietic cells mainly through activation of the PI3K/Akt pathway [10]. The inhibition of GSK3β was shown to be required for etoposide to activate the Chk1 kinase to induce G2/M cell cycle arrest and to attenuate apoptosis. However, it has remained to be examined whether other mechanisms may also be involved in synergistic induction of apoptosis by DNA damage stress and inhibition of Jak2 signaling. It is speculated that Jak2-V617F may also confer resistance to chemotherapeutic agents on hematopoietic cells through the identical mechanisms, which may be abrogated by inhibiting the aberrant kinase activity. Because several Jak2 inhibitors have been developed and under clinical evaluation as therapeutic agents for myeloproliferative neoplasms with only limited success [11], the combined effects of Jak2 inhibitors and DNA-damaging chemotherapeutic agents and the mechanisms involved in possible synergy are warranted to be studied in detail to develop effective therapeutic strategies for these diseases.

In the present study, we examine the possible effect of DNA damage stress on Jak2 and Jak2-V617F signaling in hematopoietic cells. We find that when the PI3K/Akt pathway is inhibited, GSK3β is activated by DNA damage stress and plays a role in downregulation of Jak2 and Jak2-V617F and in synergistic induction of apoptosis. Furthermore, we show that the downregulation of Jak2 and Jak2-V617F is mediated by cleavage by caspases as well as through the ubiquitin proteasome system involving the E3 ubiquitin ligases c-Cbl and Cbl-b.

## Results

### Etoposide as well as doxorubicin downregulates Jak2 and Jak2-V617F when they are inactivated

**Figure 1:**
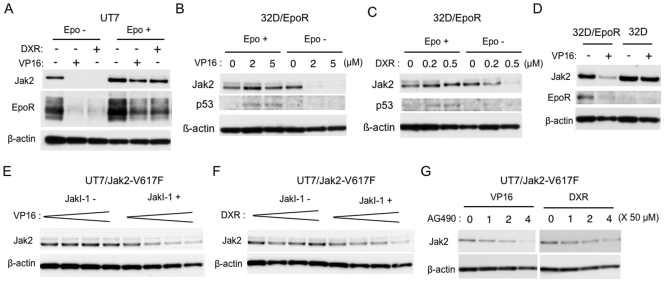
Etoposide as well as doxorubicin downregulates Jak2 and Jak2-V617F when they are inactivated. (A) After cultured for 9 h in medium without Epo, UT7 cells were left untreated or treated with 5 µM etoposide (VP16) or 0.5 µM doxorubicin (DXR) for 4 hr in the absence or presence of 50 mU/ml Epo, as indicated. Cells were lysed and subjected to immunoblot analysis with anti-Jak2 antibody, followed by reprobing with anti-EpoR and anti-β-actin, as indicated. (B, C) After cultured for 3 h in medium without Epo, 32D/EpoR cells were treated for 5 h in the absence or presence of 100 mU/ml Epo, as indicated, with increasing concentrations of etoposide (C) or doxorubicin (D), as indicated. Cell lysates were analyzed by immunoblotting with antibodies against indicated proteins. (D) 32D/EpoR or parental 32Dcl3 cells, cultured in medium containing 10% WEHI conditioning medium as the source of IL-3, were washed out of cytokine for 1 h. Cells were further cultured with or without 5 µM etoposide (VP16) for 6 h, as indicated, and analyzed. (E, F) After cultured for 9 h in medium without Epo, UT7/Jak2-V617F cells were treated for 1 h with or without 2 µM JakI-1. Cells were subsequently treated with increasing concentrations of etoposide (E; 0, 1, 2, 5 µM) or doxorubicin (D; 0, 0.1, 0.2, 0.5 µM), as indicated, and analyzed. (G) UT7/Jak2-V617F cells starved from Epo were pretreated with indicated concentrations of AG490 for 1 h. Cells were then treated with 5 µM etoposide or 0.5 µM doxorubicin for 6 h, as indicated, and analyzed.

To study the possible effect of DNA damage stress on Jak2 stability, a human leukemic cell line, UT7 [12], which expresses the wild-type Jak2 and endogenous EpoR, was treated with etoposide or doxorubicin and examined by immunoblot analysis. As shown in Fig. 1A, etoposide or doxorubicin nearly eliminated the expression level of Jak2 as well as EpoR in the absence of Epo, but not in its presence. We next examined the effects of various concentrations of etoposide as well as doxorubicin on Jak2 in a murine hematopoietic cells line, 32D/EpoR, which also expresses wild-type Jak2 and heterologously expressed EpoR [13]. As shown in Fig. 1B and 1C, etoposide as well as doxorubicin dose-dependently decreased the expression of Jak2 only when cells were starved of Epo. It was also observed that p53, a major player in DNA-damage signaling, was significantly induced by etoposide or doxorubicin only in the presence of Epo. To address the possibility that EpoR may influence the effect of DNA damage stress on Jak2, we then examined the effects of etoposide on Jak2 comparatively in 32D/EpoR and in the parental IL-3-dependent 32Dcl3 cells. As shown in Fig. 1D, when these cells were cultured with and subsequently deprived of IL-3, etoposide significantly decreased the Jak2 expression in 32D/EpoR but not in 32Dcl3 cells, thus suggesting that the presence of EpoR may facilitate the decline in Jak2 expression. Next, we examined UT7 cells expressing the constitutively activated Jak2-V617F mutant. Even in the absence of Epo, neither etoposide nor doxorubicin significantly downregulated Jak2 in UT7/Jak2-V617F cells unlike in parental UT7 cells (Fig. 1A, 1E and 1F). However, when the kinase activity was inhibited by the Jak2 inhibitor JakI-1, etoposide as well as doxorubicin dose-dependently decreased the Jak2 expression in UT7/Jak2-V617F. It was also observed that the Jak2 inhibitor AG490 dose-dependently suppressed the Jak2 expression in UT7/Jak2-V617F treated with etoposide or doxorubicin (Fig. 1G). Together, these data suggest that DNA damage stress may induce a decline in expression level of Jak2 or Jak2-V617F selectively in cells in which the kinase is in inactive state and preferentially in cells that express EpoR.

### Involvement of the PI3K/Akt/GSK3β pathway in downregulation of Jak2 in response to DNA damage

**Figure 2:**
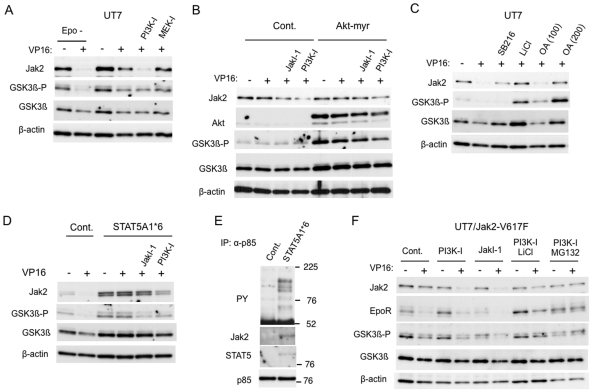
Involvement of the PI3K/Akt/GSK3β pathway in downregulation of Jak2 in response to DNA damage. (A) After cultured for 9 h in medium without Epo, UT7 cells were pretreated for 1 h with 50 µM LY294002 (PI3K-I) or 50 µM PD98059 (MEK-I), as indicated, or left untreated. Cells were subsequently treated with or without 10 µM etoposide (VP16) for 4 h, as indicated, in the presence of 20 mU/ml Epo or in its absence (Epo -). Cells were lysed and subjected to immunoblot analysis with anti-Jak2 antibody, followed by sequential reprobing with anti-phospho-GSK3α/β-S9/21 (GSK3β-P), anti-GSK3β, anti-β-actin, as indicated. (B) 32D/Akt-myr (Akt-myr) as well as control 32D/RevTRE (Cont.) cells were cultured for 24 h with 1 µg/ml doxycycline to induced the expression of Akt-myr in 32D/Akt-myr cells and subsequently washed out of WEHI conditioning medium for 12 h. Cells were then pretreated for 1 h with 1 µM JakI-1 or 10 µM LY294002 (PI3K-I), as indicated, or left untreated. Cells were finally treated with or without 10 µM etoposide (VP16), as indicated, for 4 h before analysis with indicated antibodies. (C) After cultured for 9 h in medium without Epo, UT7 cells were pretreated for 1 h with 10 µM SB216763 (SB216), 40 mM LiCl, or okadaic acid at 100 nM (OA100) or 200 nM (OA200), as indicated, or left untreated. Cells were subsequently treated with or without 10 µM etoposide (VP16) for 4 h, as indicated, and analyzed. (D) 32DE/STAT5A1*6 (STAT5A1*6) or control 32DE/pMX (Cont.) cells were pretreated for 1 h with 1 µM JakI-1 or 50 µM LY294002 (PI3K-I), as indicated, or left untreated in the absence of Epo. Cells were further treated with or without 5 µM etoposide (VP16) for 6 h, as indicated, before analysis. (E) 32DE/STAT5A1*6 (STAT5A1*6) or control 32DE/pMX (Cont.) cells were cultured overnight in the absence of Epo. Cells were lysed and subjected to immunoprecipitation of p85. Immunoprecipitates were analyzed by immunoblotting. (F) After cultured for 12 h in medium without Epo, UT7/Jak2-V617F cells were pretreated for 1 h with 50 µM LY294002 (PI3K-I), 2 µM JakI-1, 40 mM LiCl, or 10 µM MG132, as indicated, or left untreated as control (Cont.). Cells were subsequently treated with or without 5 µM etoposide (VP16), as indicated, for 6 h and analyzed.

To elucidate the mechanisms by which DNA damage stress selectively decreases the expression level of inactive Jak2, we next examined the possible involvement of signaling pathways downstream of Jak2. As shown in Fig. 2A, the PI3K inhibitor LY294002, but not the MEK inhibitor PD98059, significantly accelerated the etoposide-induced downregulation of Jak2 in UT7 cells cultured with Epo. It was also noted that treatment of cells with etoposide or doxorubicin significantly reduced the inhibitory phosphorylation of GSK3β, a target of Akt activated downstream of PI3K, particularly in the absence of Epo (Fig. 2A). These observations raise a possibility that activation of the PI3K pathway leading to Akt-mediated inactivation of GSK3β may confer the resistance on Jak2. To examine this possibility, we next comparatively examined 32D/Akt-myr cells, which express the constitutively activated Akt mutant, and the vector-control 32D/RevTRE cells. Because these cells do not express EpoR and, thus, are resistant to DNA damage stress-induced decline in Jak2 expression, we pretreated these cells with JakI-1 or with LY294002 before treating with etoposide in the absence of IL-3. As shown in Fig. 2B, pretreatment with these inhibitors allowed etoposide to decrease the Jak2 expression more significantly in control cells than in cells expressing Akt-myr, thus supporting the idea that Akt activated downstream of PI3K may confer the resistance on Jak2. We next examined the involvement of GSK3β by using its inhibitors, LiCl and SB216763, and found that these inhibitors prevented the decrease in Jak2 expression in UT7 cells treated with etoposide in the absence of Epo (Fig. 2C). Intriguingly, the PP2A inhibitor okadaic acid, previously reported to inactivate GSK3β [14], also prevented the decrease in both Jak2 and the inhibitory phosphorylation of GSK3β in a dose-dependent manner.

We also examined the possible involvement of STAT5 activation, one of the most critical Jak2 downstream signaling events, in Jak2 downregulation under DNA damage stress. For this purpose, we expressed a constitutively activated STAT5 mutant, STAT5A1*6 [15], in 32D/EpoR and examined the response to etoposide. As shown in Fig. 2D, etoposide treatment in the absence of Epo downregulated Jak2 expression in vector-control cells but not in cells expressing STAT5A1*6, which expressed Jak2 at a higher level than that in control cells. Intriguingly, the inhibitory phosphorylation of GSK3β was enhanced and was not inhibited by etoposide in cells expressing STAT5A1*6. However, Jak2 was downregulated when cells were treated with etoposide in the presence of LY294002 or, to a lesser extent, JakI-1, which also inhibited the inhibitory phosphorylation of GSK3β. To address the possible mechanisms involved in inhibition of GSK3β by STAT5A1*6, we immunoprecipitated and examined the regulatory p85 component of PI3K. Fig. 2E demonstrates that STAT5 as well as various tyrosine phosphorylated proteins are physically associated with p85 in cells expressing STAT5A1*6 but not in control cells. This is in accordance with a previous report that the PI3K/Akt pathway is activated by STAT5A1*6 through formation of molecular complexes containing p85 and STAT5 [16]. Thus, these data suggest that STAT5A1*6, or possibly activated endogenous STAT5, may inhibit GSK3β through the PI3K/Akt pathway to prevent the Jak2 downregulation under DNA damage stress.

We next examined whether Jak2-V617F is regulated similarly with wild-type Jak2 under DNA damage stress. As shown in Fig. 2F, pretreatment of UT7/Jak2-V617F cells with LY294002 as well as JakI-1 allowed etoposide to decrease the expression level of Jak2, which was prevented by co-treatment with LiCl, thus suggesting that Jak2-V617F as well as cytokine-stimulated Jak2 prevents its downregulation under DNA damage stress by activating the PI3K/Akt pathway to inhibit GSK3β activation.

### Possible involvement of ubiquitin-proteasome pathway and Cbls in downregulation of Jak2

**Figure 3:**
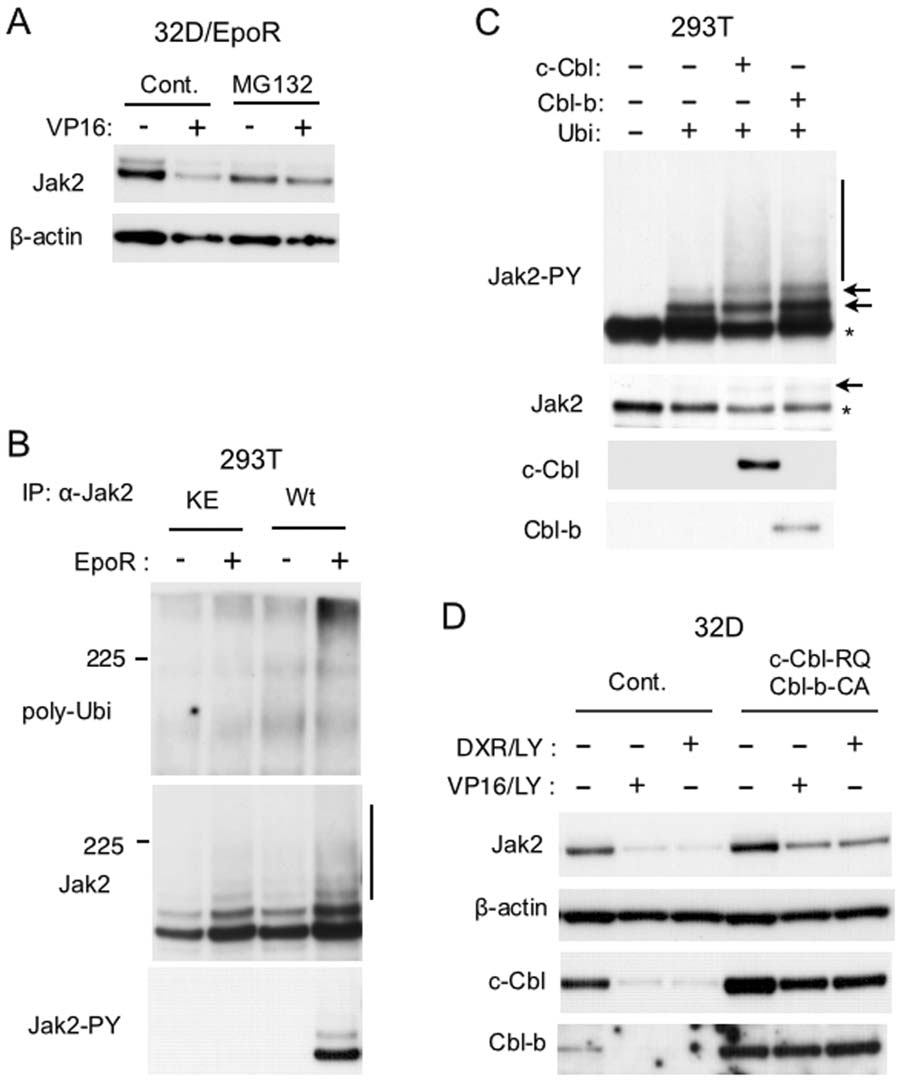
Possible involvement of ubiquitin-proteasome pathway and Cbls in downregulation of Jak2. (A) 32D/EpoR cells deprived of Epo for 2 h were pretreated with 10 µM MG132 or left untreated as control, as indicated for 1 h in the absence of Epo. Cells were then treated for 6 h with or without 5 µM etoposide, as indicated. Cells were lysed and subjected to immunoblot analysis using indicated antibodies. (B) 293T cells were transfected on 6-well plate with 0.1 µg of pRK5-Ubiquitin-WT and 0.002 µg of pRK5-Jak2-Wt (Wt) or pRK5-Jak2-KE (KE) along with 0.1 µg of pXM-EpoR-Wt or empty plasmid, as indicated. Two days after transfection, cells were lysed, and Jak2 was immunoprecipitated. Immunoprecipitates were analyzed by immunoblotting using antibodies against polyubiquitin (poly-Ubi), Jak2, and Jak2 phosphorylated on Y1007 (Jak2-PY), as indicated. The vertical line indicates the smeary pattern characteristic of ubiquitination. (C) 293T cells were transfected on 6-well plate with 0.3 µg of pRevTRE-Jak2V617F and 0.2 µg of pTet-On along with 0.2 µg of pRK5-Ubiquitin-WT (Ubi), 0.5 µg of pRevTRE-c-Cbl (c-Cbl), or 0.5 µg of pRevTRE-Cbl-b (Cbl-b), as indicated. The amounts of plasmid DNA transfected were equalized by adding pRevTRE. Two days after transfection, cells were lysed, and lysates were analyzed by immunoblotting. The position of unmodified Jak2-V617F and those of ubiquitinated Jak2-V617F are indicated by asterisks and arrows, respectively. (D) Ton.32D/Flt3-Wt (Cont.) and Ton.32D/Flt3-Wt/c-Cbl-RQ/Cbl-b-CA (c-Cbl-RQ/Cbl-b-CA) cells, cultured in doxycycline-containing medium, were cultured for 3 h with or without 5 µM LY294002 (LY), as indicated, in the absence of IL-3. Cells were further treated for 6 h with 10 µM etoposide (VP16) or 1 µM doxorubicin (DXR), as indicated, and lysed. Cell lysates were analyzed by immunoblotting.

To address the possibility that Jak2 may undergo degradation in cells under DNA damage stress, we pretreated 32D/EpoR cells with the proteasome inhibitor MG132 before treatment with etoposide. As shown in Fig. 3A, the decrease in Jak2 expression in these cells was prevented by MG132, which also prevented the decline in Jak2-V617 in cells treated with both LY294002 and etoposide (Fig. 2F). These data suggest that Jak2 as well as Jak2-V617F may be degraded at least partly through the proteasome pathway in response to DNA damage stress unless GSK3β is inactivated downstream of the PI3K/Akt pathway.

Because ubiquitination plays a critical role in regulation of proteasomal degradation of various proteins including tyrosine kinases [17], we studied ubiquitination of Jak2 transiently expressed with ubiquitin in 293T cells. As shown in Fig. 3B, wild-type Jak2 but not a kinase-dead Jak2 mutant was significantly polyubiquitinated when co-expressed with EpoR. In accordance with our previous report, Jak2 becomes activated and autophosphorylated on Y1007 in the activation loop without Epo stimulation when overexpressed with EpoR [18]. Thus, these data are also in accordance with a previous report that the phosphorylation on Y1007 is critical for polyubiquitination of Jak2 [19]. On the other hand, Jak2-V617F was constitutively phosphorylated on Y1007 without EpoR and was ubiquitinated when coexpressed with ubiquitin in 293T cells, which was detected by immunoblotting with anti-phospho-Y1007-Jak2 antibody as additional slowly migrating bands and the typical smeary pattern (Fig. 3C). Intriguingly, ubiquitination of Jak2-V617F was significantly enhanced by co-expressing c-Cbl or Cbl-b, the E3 ubiquitin ligase known to play important roles in regulation of degradation of various tyrosine kinases through the ubiquitin proteasome pathway as well as in leukemogenesis [20], [21].

To address the possibility that the Cbl ubiquitin ligases may be involved in degradation of Jak2 under DNA damage stress, we next examined 32D cells that overexpress both c-Cbl-R420Q and Cbl-b-C373A, loss-of-function mutants of the ubiquitin ligases. As shown in Fig. 3D, the decline in Jak2 expression induced by treatment with etoposide or doxorubicin in the presence of LY294002 was significantly attenuated by overexpression of these putative dominant negative mutants. These data strongly implicate c-Cbl as well as Cbl-b in regulation of Jak2 degradation under DNA damage stress.

### Caspase activation is involved in Jak2 degradation in cells under DNA damage stress

**Figure 4:**
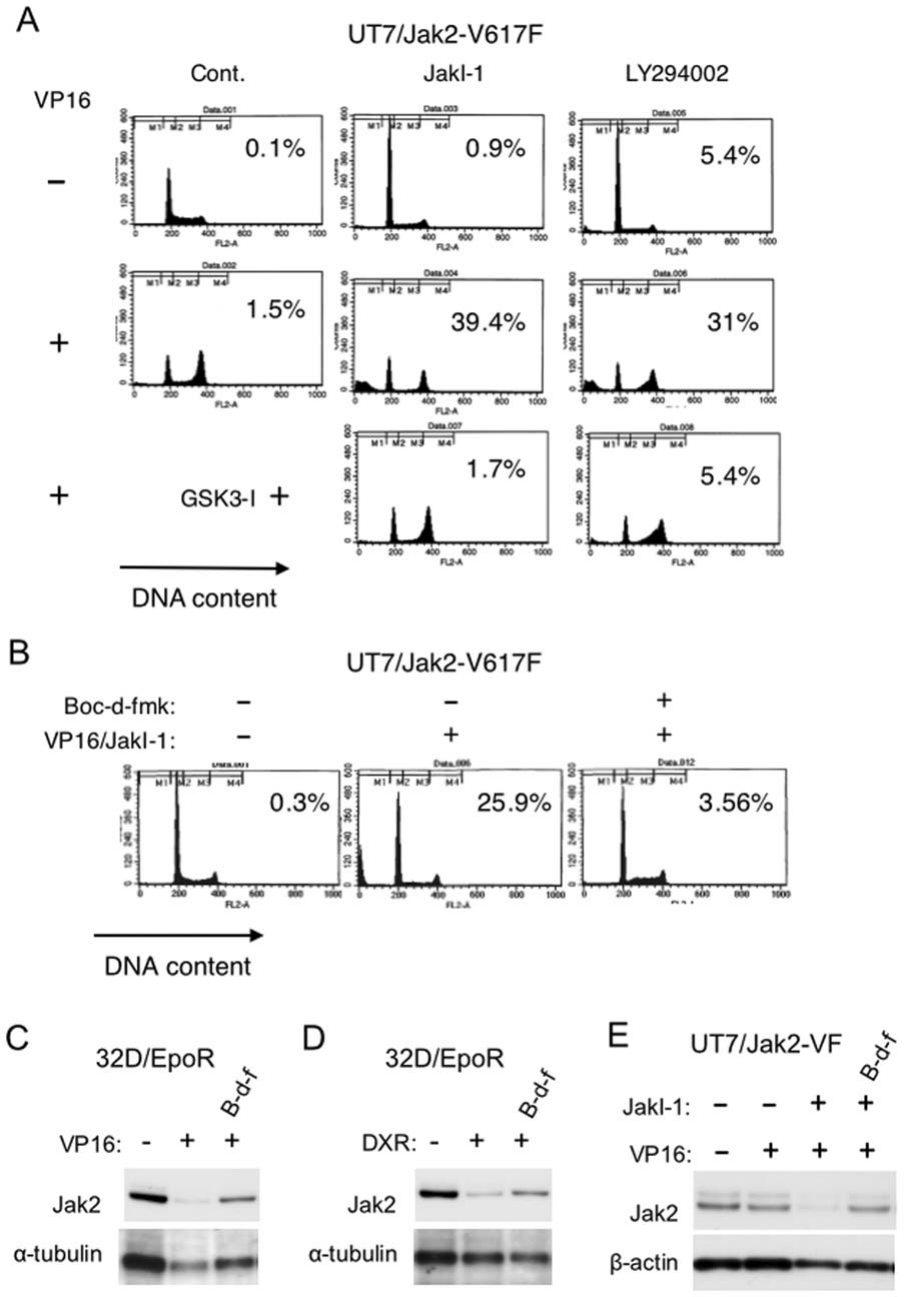
Synergistic induction of apoptosis in UT7/Jak2-V617F by etoposide and JakI-1 and effects of Boc-d-fmk on Jak2 downregulation. (A) UT7/Jak2-V617F cells were cultured for 12 h with 0.5 µM etoposide (VP16), 0.5 µM JakI-1, 25 µM LY294002, and 1 µM GSK3I-5, as indicated in the absence of Epo, and analyzed for the cellular DNA content by flow cytometry. Percentages of apoptotic cells with sub-G1 DNA content are indicated. (B) UT7/Jak2-V617F cells were cultured for 6 h with 5 µM etoposide (VP16), 1 µM JakI-1, and 100 µM Boc-d-fmk, as indicated, in the absence of Epo and analyzed for the cellular DNA content. (C, D) 32D/EpoR cells deprived of Epo for 2 h were pretreated for 1 h with 100 µM Boc-d-fmk (B-d-f), as indicated, or left untreated. Cells were then treated for 5 h with or without 5 µM etoposide (VP16) or 0.5 µM doxorubicin (DXR), as indicated. Cells were lysed and analyzed by immunoblotting. (E) UT7/Jak2-V617F cells, cultured without Epo for 12 h, were treated with 1 µM JakI-1 and 100 µM Boc-d-fmk (B-d-f), as indicated, or left untreated. Cells were then treated with or without 5 µM etoposide (VP16), as indicated, for 6 h and analyzed.

We have previously shown that inhibition of the PI3K pathway by LY294002 or by cytokine deprivation synergistically enhanced etoposide-induced apoptosis of hematopoietic cells, including 32D and UT7, at least partly by inhibiting the Chk1-mediated G2/M checkpoint activation [10]. To examine whether similar synergistic effects could be observed in UT7 cells expressing Jak2-V617F, we treated these cells with etoposide and JakI-1 or LY294002. Treatment of UT7/Jak2-V617F cells with etoposide alone for 12 h induced G2/M arrest without significantly inducing apoptosis. However, cotreatment with JakI-1 or LY294002 synergistically enhanced etoposide-induced apoptosis as detected by cells with sub-G1 DNA content, which was prevented by inhibiting GSK3β by adding the specific inhibitor GSK3I-5 or LiCl (Fig. 4A and data not shown). Thus, apoptosis induced by etoposide in combination with these inhibitors correlated with the decline in Jak2-V617F expression, which raises the possibility that activation of caspases may be involved in degradation of Jak2-V617F as well as Jak2.

To address this possibility, we utilized the pan-caspase inhibitor Boc-d-fmk, which effectively prevented apoptosis induced by combined treatment of UT7/Jak2-V617F cells with etoposide and JakI-1 (Fig. 4B). As shown in Fig. 4C and 4D, Boc-d-fmk partially protected Jak2 from degradation in 32D/EpoR cells treated with etoposide or doxorubicin in the absence of Epo. Similarly, Boc-d-fmk protected degradation of Jak2-V617F in UT7/Jak2-V617F cells treated with both JakI-1 and etoposide (Fig. 4E).

**Figure 5:**
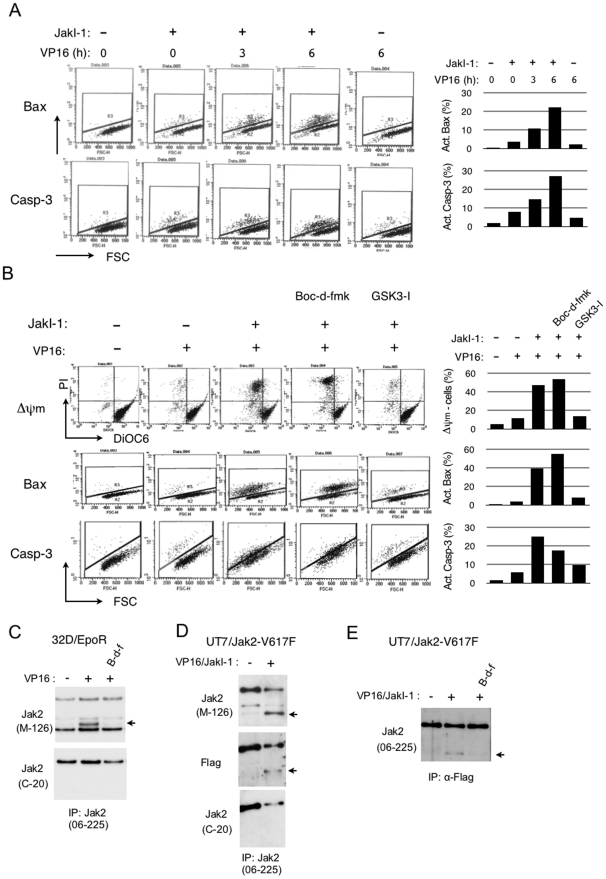
Caspase activation is involved in Jak2 degradation in cells under DNA damage stress. (A) UT7/Jak2-V617F cells, cultured without Epo, were treated for 7 h with or without 1 µM JakI-1, as indicated, with 1 µM etoposide (VP16) added for indicated times before harvest. Cells were then analyzed for activation of Bax or caspase-3 by flow cytometry. Percentages of cells with activated Bax or caspase-3 are plotted. FSC: forward scatter. (B) UT7/Jak2-V617F cells, cultured without Epo, were pretreated for 1 h with 1 µM JakI-1, 100 µM Boc-d-fmk, or 1 µM GSK3I-5, as indicated. Cells were further treated for 6 h with or without 5 µM VP16 and analyzed for loss of mitochondrial membrane potential and activation of Bax or caspase-3, as indicated, by flow cytometry. (C) 32D/EpoR cells were deprived of Epo and pretreated with 100 µM Boc-d-fmk (B-d-f), as indicated, or left untreated. Cells were then treated for 5 h with or without etoposide (VP16), as indicated, before harvest. Immunoprecipitates obtained using anti-Jak2 (06–255) were analyzed by immunoblotting using anti-Jak2 (M-126) and anti-Jak2 (C-20), as indicated. The numbers of cells treated with etoposide were 2.5 times that of cells untreated. An arrow indicates the 100-kDa band. (D) UT7/Jak2-V617F cells, cultured without Epo, were pretreated for 1 h with or without 1 µM JakI-1, as indicated. Cells were then treated for 8 h with or without 5 µM etoposide (VP16), as indicated, before harvest. Immunoprecipitates obtained using anti-Jak2 (06–255) were analyzed by immunoblotting using indicated antibodies. The number of cells treated with etoposide was 2 times that of cells untreated. (E) UT7/Jak2-V617F cells, cultured without Epo, were pretreated for 1 h with 1 µM JakI-1 and 20 µM Boc-d-fmk (B-d-f), as indicated. Cells were then treated for 6 h with or without 5 µM etoposide (VP16), as indicated, before harvest. Immunoprecipitates obtained using anti-Flag was were analyzed by immunoblotting using anti-Jak2 (06–255). The numbers of cells treated with etoposide were 2.5 times that of cells untreated.

To confirm the relevant caspase activation in these cells, we next examined by flow-cytometry the expression level in UT7/Jak2-V617F cells of cleaved and, thus, activated caspase-3 as well as that of Bax in activated conformation, which acts upstream of mitochondria to initiate the intrinsic apoptotic signaling pathways leading to activation of the executioner caspase, caspase-3 [9]. As shown in Fig. 5A, treatment with etoposide or JakI-1 alone for 6 h or 7 h, respectively, only marginally activated Bax and caspase-3. However, in the presence of JakI-1, etoposide began to activate Bax and caspase-3 as early as 3 h after treatment and significantly activated these molecules after 6 h of treatment. These data indicate that etoposide and JakI-1 synergistically and rapidly activate the Bax-mediated apoptotic pathway leading to the cleavage of caspase-3. Thus, the activation of caspase-3 and the degradation of Jak2-V617F correlated in UT7 cells treated with JakI-1 and etoposide. We next examined the effects of Boc-d-fmk and GSK3I-5 on apoptosis-inducing events in these cells. As expected, JakI-1 drastically enhanced etoposide-induced decline of mitochondrial membrane potential, the downstream event of Bax activation, which leads to the activation of caspase cascade (Fig. 5B). Boc-d-fmk partially inhibited the cleavage of caspase-3 without showing any inhibitory effects on activation of Bax and decline of mitochondrial membrane potential, which is in agreement with the idea that the latter events are upstream of the former. Intriguingly, the GSK3β inhibitor remarkably inhibited activation of Bax as well as decline of mitochondrial membrane potential, thus significantly reducing the caspase-3 activation. Therefore, the effects of these inhibitors on caspase-3 activation correlated with those on Jak2-V617F degradation. These data also indicate that activation of GSK3β may play a critical role in synergistic activation of Bax by the combined treatment with JakI-1 and etoposide.

Finally, we examined whether cleaved fragments of Jak2 could be detected after caspase activation in cells under DNA damage stress. 32D/EpoR cells deprived of Epo were treated with etoposide, and Jak2 was immunoprecipitated with an antibody against the Jak2 amino acids 758–776, anti-Jak2 (06–255), and immunoblotted with an antibody against Jak2 amino acids 190–315, anti-Jak2 (M-126). Although a band of about 95 kDa was constantly observed in addition to the 130-kDa band corresponding to full-length Jak2 in all the samples, a band of about 100 kDa was conspicuously observed only in a sample from cells treated with etoposide but not from cells left untreated or treated with both etoposide and Boc-d-fmk (Fig. 5C). These additional bands were not observed when an antibody against the C-terminal polypeptide of Jak2, anti-Jak2 (C-20), was used for immunodetection. This antibody also failed to detect the expected smaller C-terminal fragment of Jak2. Similarly, treatment of UT7/Jak2-V617F cells with both JakI-1 and etoposide produced an extra band of about 100 kDa that was immunoprecipitated with anti-Jak2 (06–255) and detected by anti-Jak2 (M-126) but not by anti-Jak2 (C-20). This band was also detected by antibody against the Flag epitope, which was tagged at the N-terminus of Jak2-V617F. Conversely, cell lysates were subjected to immunoprecipitation with anti-Flag and probed with anti-Jak2 (16–255). As expected, the 100-kDa band was detected only when cells were treated with both etoposide and JakI-1 but not when they were similarly treated in the presence of Boc-d-fmk or left untreated (Fig. 5E). These data suggest that Jak2 as well as Jak2-V617 may be cleaved at a site C-terminal to amino acids 758–776 directly or indirectly by activated caspases under DNA damage stress when the kinase activity is inactive.

## Discussion

We have demonstrated in the present study that GSK3β should play a crucial role in synergistic induction of apoptosis in hematopoietic cells treated with DNA-damaging agents under conditions where Jak2 is inhibited, because GSK3β inhibitors prevented the apoptosis (Fig. 4A and data not shown). Under these conditions, the PI3K/Akt pathway downstream of Jak2 is downregulated, thus reducing the inhibitory phosphorylation of GSK3β. According to previous reports, GSK3β is also activated by etoposide or ceramide, which may be mediated indirectly through PP2A activation [14], [22], [23]. In accordance with this, etoposide or doxorubicin reduced the inhibitory phosphorylation of GSK3β, which was inhibited by the PP2A inhibitor okadaic acid (Fig. 2C). Thus, it is speculated that, possibly through activation of PP2A, DNA damage stress in combination with inactivation of the PI3K/Akt pathway may activate GSK3β beyond a threshold level where apoptosis is imminent. We have previously implicated GSK3β activation in inhibition of Chk1-mediated G2/M checkpoint activation leading to synergistic induction of apoptosis in hematopoietic cells treated with etoposide in the absence of Epo [10]. However, the synergistic activation of Bax and caspase-3 by etoposide and JakI-1 in UT7/Jak2-V617F was observed as early as 3 h after treatment (Fig. 5A), thus suggesting that molecular mechanisms other than that regulating cell cycle progression may also play an important role in activation of apoptotic signaling downstream of GSK3β. In this regard, GSK3β has been implicated in direct or indirect activation of Bax, caspase-2, and caspase-3 leading to apoptosis [14], [23], [24]. These possibilities as well as the mechanisms involved in GSK3β activation under DNA damage stress need to be addressed in future studies.

The present study has implicated the ubiquitin proteasome pathway in degradation of Jak2 under DNA stress, because the proteasome inhibitor MG132 prevented the decline in Jak2 (Fig. 3A). Moreover, transient expression studies showed that Jak2 is polyubiquitinated when co-expressed with EpoR and autophosphorylated on Y1007 (Fig. 3B). This is in agreement with the previous report that Jak2 becomes polyubiquitinated when autophosphorylated on Y1007 [19]. However, it also raises a possibility that EpoR may play a role in polyubiquitination of Jak2 independent of its activation, which might explain the fact that Jak2 was downregulated much more significantly in cells expressing EpoR (Fig. 1D). Furthermore, the E3 ubiquitin ligases c-Cbl and Cbl-b enhanced ubiquitination of Jak2-V617F in 293T cells (Fig. 3C) and were implicated in degradation of Jak2 under DNA damage stress in 32D cells, because overexpression of loss-of-function mutants of both c-Cbl and Cbl-b impeded the degradation (Fig. 3D). This may bear clinical relevance for treatment of myeloproliferative neoplasms, because these mutants of Cbls have been observed in these diseases [21]. It should be noted, however, that in most cases the degradation of tyrosine kinases through the ubiquitin proteasome system is triggered upon its activation and autophosphorylation, which may create a binding site for an E3 ligase [17]. In accordance with this, it was reported that autophosphorylation of Jak2 on Y1007 creates a binding site for the SOCS1 complex, resulting in ubiquitination and proteasomal degradation of Jak2 [19]. It was more recently reported that Jak2-V617F constitutively undergoes SOCS1- and SOCS3-mediated polyubiquitination and proteasomal degradation [25]. In this report, JakI-1 was shown to inhibit the degradation and to increase the expression level of Jak2-V617F. In contrast with these reports, Jak2 is dephosphorylated on Y1007 when cells are deprived of Epo, and JakI-1 allowed DNA damage to degrade Jak2-V617F in the present study. It should be also noted that we could not detect significant polyubiquitination of Jak2 or Jak2-V617F when they were downregulated in hematopoietic cells in response to DNA damage stress (negative data not shown). Thus, the possibilities remain that MG132 and c-Cbl or Cbl-b may affect the Jak2 downregulation through indirect mechanisms, which needs to be addressed in future studies.

It has been reported that a Jak2 inhibitor, WP1066, induced Jak2 degradation through a proteolytic mechanism that was not inhibited by proteasome inhibitors [26], [27]. A histone deacetylase inhibitor, panobinostat, was also reported to downregulate the Jak2-V617F expression, which was most likely through inhibition of HSP90 by panobinostat [28]. A more recent report showed that an HSP90 inhibitor, PU-H71, downregulated the expression of Jak2 as well as Jak2-V617F and reduced viability of cells [29]. In this report, it was found that HSP90 physically associated with Jak2 or Jak2-V617F, which was independent of its phosphorylation status. Similarly with the present study, the downregulation was inhibited by MG132. The authors thus concluded that HSP90 protects Jak2 and Jak2-V617F from proteasomal degradation, although the possible involvement of caspases or other proteolytic enzymes was not addressed. Therefore, it is possible that the interaction with HSP90 may be involved in the downregulation of Jak2 and Jak2-V617F in the present study. This possibility needs to be addressed thoroughly in future studies. However, our preliminary data suggest that Jak2 inhibition under DNA damage stress dose not significantly affect its interaction with HSP90.

The tumor suppressor p53 plays a major role in DNA-damage stress signaling to control cell cycle progression, DNA repair, and apoptosis [30]. Intriguingly, Nakatake et al. [31] very recently reported that Jak2-V617F inhibited the induction of p53 expression by DNA-damage stress. Unfortunately, we could not examine the effects of Jak2-V617F expression and its inhibition on DNA damage-induced expression of p53 in UT7 cells, because these cells lack the normal *TP53* transcript [32]. On the other hand, as shown in Fig. 1B and 1C, the induction of p53 by etoposide or doxorubicin was inhibited when Jak2 was inactivated by Epo withdrawal in 32D/EpoR cells. This may be at least partly due to the Epo-dependence of Chk1 activation in these cells [10], which plays a role in induction of p53 [30]. Irrespective of the mechanisms involved in modulation of the p53 induction by Jak2 or the V617F mutant, p53 may not play a significant role in DNA damage-induced degradation of Jak2, which was observed similarly in cells expressing p53 or not.

Recent studies have shown that caspase-mediated cleavage of kinases can terminate prosurvival signaling or generate proapoptotic peptide fragments that help to execute the death program [33]. Because the cleavage of Jak2 or Jak2-V617F occurs early in the apoptotic process, it is tempting to speculate that this cleavage may play an important role in the synergistic induction of apoptosis under DNA damage stress. Judging from the size of putative cleavage product (100 kDa), which reacts with anti-Jak2 (06–255) against the Jak2 amino acids 758–776, the cleavage site should be located within the tyrosine kinase (JH1) domain or between the JH1 and the pseudokinase (JH2) domains, the latter of which possesses the inhibitory effect on the kinase activity [1]. Thus, caspases may abolish the Jak2 kinase activity by cleaving it within the JH1 domain to terminate prosurvival signaling. Alternatively, it is also possible that the cleavage between the JH1 and JH2 domains create an active tyrosine kinase domain fragment that may play a proapoptotic or possibly prosurvival role, although we could not detect the smaller C-terminal fragment of Jak2 using anti-Jak2 (C-20). We are currently trying to identify the cleavage site by mutagenesis of the putative caspase cleavage sites and to determine the significance of Jak2 and Jak2-V617F cleavage on the synergistic induction of apoptosis.

Although several Jak2 inhibitors have been developed and under clinical evaluation, the therapy of patients with myeloproliferative neoplasms with these agents has thus far shown only modest effects at best [11]. In this regard, the present study may contribute to the development of possible combination therapies of Jak2 inhibitors and DNA-damaging chemotherapeutic agents.

## Materials and Methods

### Cells and reagents

A murine IL-3-dependent cell line, 32Dcl3, and a clone of 32Dcl3 expressing the Epo receptor, 32D/EpoR, have been previously described [13] and maintained in RPMI1640 medium supplemented with 10% FCS and 10% WEHI conditioning medium as the source of IL-3 or 1 U/ml human recombinant Epo. Ton.32D (A clone of 32Dcl3 cells transfected with pTet-On (Clontech)), Ton.32D/Flt3-Wt (Ton.32D cells expressing Flt3), and Ton.32D/Flt3-Wt/c-Cbl-RQ/Cbl-b-CA (Flt3-expressing Ton.32D cells that overexpress loss-of-function mutants of both c-Cbl (c-Cbl-R420Q) and Cbl-b (Cbl-b-C373A) when cultured with doxycycline) have also been described (Oshikawa, G., Nagao, T., Wu, N., Kurosu, T., and Miura, O., submitted for publication). A human leukemic cell line expressing the endogenous EpoR, UT7 [12], was kindly provided by Dr N. Komatsu and maintained in RPMI1640 medium with 10% FCS and 1 U/ml Epo. PLAT-A [34], an amphotropic virus packaging cell line, and 293T, a human embryonic kidney cell line, were kindly provided by Dr. T. Kitamura and Dr. S. Yamaoka, respectively, and maintained in Dulbecco's modified Eagle's medium (DMEM) supplemented with 10% FCS.

Recombinant human Epo was kindly provided by Chugai Pharmaceutical Co. Ltd. (Tokyo, Japan). Doxycycline was purchased from Sigma (St Louis, MO, USA). Etoposide, doxorubicin, hygromycin, and LiCl were purchased from Wako (Tokyo, Japan). The PI3K inhibitor LY294002, the MEK1 inhibitor PD98059, the GSK3β inhibitor SB216763, MG132, JakI-1, AG490, and okadaic acid were purchased from Calbiochem (La Jolla, CA, USA). GSK3β-inhibitor #5 (GSK3I-5) [35] was synthesized and kindly provided by Dr. H. Kagechika. The pan-caspase inhibitor Boc-d-fmk was purchased from Biovision (Mountain View, CA, USA). DiOC6 was purchased from Invitrogen (Carlsbad, CA, USA).

Rabbit polyclonal antibodies against Akt, cleaved caspase-3, GSK3β, p85, phospho-Jak2-Y1007/1008, phospho-GSK3α/β-S9/21, and p85 as well as rabbit or mouse monoclonal antibody against phospho-STAT5-Y694 or p53 (1C12), respectively, were purchased from Cell Signaling Technology (Beverly, MA, USA). Rabbit polyclonal antibodies against c-Cbl, Cbl-b, EpoR, Jak2 (C-20 and M-126), and STAT5A as well as a mouse monoclonal antibody against α-tubulin were purchased from Santa Cruz Biotechnology (Santa Cruz, CA, USA). A rabbit antibody against Jak2 (06–255) and a mouse monoclonal antibody against phosphotyrosine (4G10) were purchased from Millipore (Billerica, MA, USA). Anti-Jak2 (06–255) was used throughout the present studies unless described otherwise. A mouse monoclonal antibody against FLAG (M2) and β-actin were purchased from Sigma. A mouse monoclonal antibody against polyubiquitin (FK-1) was purchased from Enzolifesciences (Farmingdale, NY, USA). A mouse monoclonal antibody against Bax (YTF-6A7) was purchased from Trevigen (Gaithersburg, MD, USA).

### Expression plasmids

An expression plasmid for Jak2-V617F, pMSCV-Jak2V617F [36], was kindly provided by Dr. R. Skoda. Expression plasmids, tTA-IRES-GFP (Addgene plasmid 18783) and pRK5-Ubiquitin-WT (Addgene plasmid 17608) [37], were purchased from Addgene (Cambridge, MA, USA). An Expression plasmid for murine EpoR, pXM-EpoR-Wt, was described previously [13]. Expression plasmids encoding wild-type Jak2 (pRK5-Jak2-Wt) and a kinase negative mutant Jak2 (pRK5-Jak2-KE) [38] were kindly provided by Dr. JN. Ihle. An expression plasmid, pMX-puro-STAT5A1*6 [15], was kindly provided by Dr. T. Kitamura. An expression plasmid, pUSE-myr-Akt1, was purchased from Millipore. pRevTRE-c-Cbl and pRevTRE-Cbl-b have been described [39]. pRevTRE and pTet-On were purchased from Clontech (Mountain View, CA, USA).

To prepare a Flag-tagged Jak2-V617F expression vector, the XhoI/HpaI fragment of pMSCV-Jak2V617F was subcloned into the pGEM-T-easy vector, purchased from Promega (Madison, WI, USA), thus adding the FLAG-tag coding sequence at the 5'-terminus, to give pGEM-T-easy-Jak2V617F. The SalI/SphI fragment of pGEM-T-easy-Jak2V617F was subsequently subcloned between the SalI/SphI site in the pRevTRE vector to give pRevTRE-Jak2V617F. A retroviral expression plasmid, pRevTRE-myr-Akt1, was constructed by subcloning the HindIII/PmeI fragment from pUSE-myr-Akt1 into the HindIII/HpaI site of pRevTRE.

### Transfection and infection

For retroviral infection, PLAT-A cells were first transfected with tTA-IRES-GFP using the Lipofectamin reagent (GIBCO-BRL, Grand Island, NY), according to the manufacturer's instruction. The recombinant retrovirus was harvested 48 h after transfection and used to infect UT7 cells. Cells expressing green fluorescent protein (GFP) were sorted using a Becton Dickinson FACSscan flow cytometer (Mountain View, CA, USA). Isolated cells were infected again with the pRevTRE-Flag-Jak2V617F or pRevTRE retrovirus and selected in medium containing 600 µg/ml hygromycin. Pools of infected cells were used in subsequent experiments as UT7/Jak2-V617F or UT7/RevTRE cells. Ton.32D cells were similarly infected with the pRevTRE-myr-Akt1 or pRevTRE retrovirus and selected in medium containing 600 µg/ml of hygromycin to be used as 32D/Akt-myr or 32D/RevTRE cells, respectively. 32D/EpoR cells were infected with the pMX-puro or pMX-puro-STAT5A1*6 retrovirus and selected in medium containing 1 µg/ml puromycin to be used as 32DE/pMX or 32DE/STAT5A1*6 cells, respectively.

For transient expression in 293T cells, cells were transfected with indicated plasmids using the Lipofectamine reagent. Cells were harvested two days after transfection for immunoprecipitation and immunoblotting.

### Immunoprecipitation and immunoblot analyses

For immunoprecipitation experiments, cells were lysed in a lysis buffer containing 1% Triton X-100, 20 mM Tris-HCl (pH 7.5), 150 mM NaCl, 1 mM EDTA, 1 mM sodium orthovanadate, 1 mM phenylmethylsulfonyl fluoride and 10 µg/ml each of aprotinin and leupeptin. Cell lysates were subjected to immunoprecipitation and immunoblotting as described previously [13]. For immunoblot analysis of total cell lysates, samples were prepared by mixing an aliquot of cell lysates with an equal volume of 2X Laemmli's sample buffer and heating at 100°C for 5 min. The results shown are representative of experiments repeated at least three times.

### Flow cytometric analyses of apoptosis, Bax, caspase-3, and mitochondrial membrane potential

Flow cytometric analysis of cell cycle and apoptosis was performed as described previously [40]. Flow cytometric analyses of the Bax conformational change and caspase-3 cleavage were performed using specific antibodies against activated Bax and cleaved caspase-3 as described previously [41]. For analysis of mitochondrial membrane potential, cells were stained with a lipophilic cation (5, 5', 6, 6', tetrachloro-1, 1', 3, 3'-tetraethylbenzimidazolyl carbocyanin iodide) using the DePsipher kit (Trevigen) and analyzed by flow cytometry as described previously [42].
